# Protein refolding based on high hydrostatic pressure and alkaline pH: Application on a recombinant dengue virus NS1 protein

**DOI:** 10.1371/journal.pone.0211162

**Published:** 2019-01-25

**Authors:** Rosa Maria Chura-Chambi, Cleide Mara Rosa da Silva, Lennon Ramos Pereira, Paolo Bartolini, Luis Carlos de Souza Ferreira, Ligia Morganti

**Affiliations:** 1 Centro de Biotecnologia, Instituto de Pesquisas Energéticas e Nucleares, IPEN-CNEN/SP, São Paulo, São Paulo, Brazil; 2 Departamento de Microbiologia, Instituto de Ciências Biomédicas, Universidade de São Paulo, São Paulo, São Paulo, Brazil; Stanford University School of Medicine, UNITED STATES

## Abstract

In this study we evaluated the association of high hydrostatic pressure (HHP) and alkaline pH as a minimally denaturing condition for the solubilization of inclusion bodies (IBs) generated by recombinant proteins expressed by *Escherichia coli* strains. The method was successfully applied to a recombinant form of the dengue virus (DENV) non-structural protein 1 (NS1). The minimal pH for IBs solubilization at 1 bar was 12 while a pH of 10 was sufficient for solubilization at HHP: 2.4 kbar for 90 min and 0.4 kbar for 14 h 30 min. An optimal refolding condition was achieved by compression of IBs at HHP and pH 10.5 in the presence of arginine, oxidized and reduced glutathiones, providing much higher yields (up to 8-fold) than association of HHP and GdnHCl via an established protocol. The refolded NS1, 109 ± 9.5 mg/L bacterial culture was recovered mainly as monomer and dimer, corresponding up to 90% of the total protein and remaining immunologically active. The proposed conditions represent an alternative for the refolding of immunologically active recombinant proteins expressed as IBs.

## Introduction

*Escherichia coli* strains are the most usual alternative for production of heterologous proteins, particularly for those that do not require post-translational modifications [1]. Depending on the characteristics of the expressed protein, incubation temperature and expression levels, it may be produced in soluble form or as insoluble aggregates: the inclusion bodies (IBs) [2]. In IBs the proteins form amyloid-like structures in which molecules, with a conformation that include the native ones, are trapped [3]. Indeed, proteins in IBs frequently keep secondary and tertiary structures similar to those found in their native conformation [4] and may even show some degree of biological activity [5, 6]. In addition, the expression of recombinant proteins as IBs provides recombinant proteins with very low levels of contaminants.

In contrast, the difficulties faced to obtain a fully active protein from IBs is a frequent drawback. Usually the first step in the refolding processes is the solubilization of the insoluble aggregates with high concentrations of denaturing agents, such as urea or guanidine hydrochloride (GdnHCl). After removal of the denaturing agent, a massive protein aggregation is frequently observed mainly due to intermolecular hydrophobic interactions [7]. Thus, for the purpose of solubilizing IBs, alternative milder solubilization processes have been reported avoiding disruption of native-like intramolecular bonds, such as the use of high hydrostatic pressure (HHP) and alkaline pH conditions. HHP promotes the solubilization of protein aggregates by rupture of hydrophobic and of ionic interactions [8]. However, HHP is frequently not sufficient to achieve an efficient protein solubilization and other agents, such as GdnHCl at low concentrations are frequently used [9, 10]. Another condition, used for the solubilization of IBs with low degree of protein unfolding, is alkaline pH, which breaks intermolecular bonds by electrostatic repulsion [11]. Nonetheless, efficient protein solubilization at alkaline pH also requires the concomitant use of a denaturing reagent [12–14].

In the present study, we tested the combination of HHP and alkaline pH for the refolding of recombinant proteins accumulated as IBs expressed in *E*. *coli* cytoplasm. For such purpose we tested, as a model, the dengue virus (DENV) non-structural protein 1 (NS1). NS1 is the most used antigen for diagnostic tests in acute or convalescent patients infected by flavivirus and may also have applications as a vaccine antigen [15].

Our results demonstrated that association of HHP with alkaline conditions allowed the dissociation of aggregates into immunologically active soluble NS1 at high yields and, thus, represents a novel alternative for the solubilization of recombinant proteins accumulated in IBs.

## Methods

### Expression of recombinant proteins, bacterial lysis and NS1-IBs washes

The gene that codifies for the full-length DENV NS1 (GenBank: M29095.1) with one histidine tag at the N-terminal was synthesized and cloned in the plasmid pET28A (Merck Millipor Inc) by GenScript. (USA). *Escherichia coli*, strain BL21 (DE3) (Merck Millipore Inc.), was used as host. Bacterial culture (1 L) was carried out in a rotary shaker at 37 °C in LB medium. NS1 expression induction was performed for 13 h after addition of 0.5 mM IPTG when cultures reached an optical density (at 600 nm) of 0.5, as described [16]. The culture was centrifuged at 3,000 x g for 15 minutes at 4 °C and the supernatant was discarded. The pellet was resuspended in 50 mL of buffer A1 (0.1 M Tris-HCl pH 8.5 + 5 mM EDTA), lysozyme (50 μg / mL) was added and the suspension was incubated at room temperature for 15 minutes. Sodium deoxycholate was then added to reach a concentration of 0.1%. Bacterial lysis was performed by sonicating the suspension on ice until the solution lost viscosity and was then centrifuged at 9,500 x g for 20 minutes at 4 °C. The supernatant was discarded and the pellet was resuspended in 50 mL of buffer A2 (0.1 M Tris-HCl pH 8.5 containing 5 mM EDTA and 0.1% sodium deoxycholate). The suspension was sonicated rapidly to disrupt the lumps and centrifuged at 9,500 x g for 20 minutes at 4 °C, and NS1-IBs were washed again. They were washed once with 0.1 M Tris HCl buffer at a pH of 8.5 containing 50 mM EDTA, centrifuged and resuspended in 10–20 mL of the same buffer. The absorbance was read on a spectrophotometer at 350 nm, and the IBs suspension was separated into 1 mL aliquots that were kept in a freezer (-20 °C) until use.

### High hydrostatic pressure

The optical density of the suspension of NS1-IBs was determined by spectrophotometer at 350 nm and diluted to 1.0, 2.0 or 5.0 optical units (A_600 nm_) with the appropriate buffer containing 1 mM EDTA. The buffers used were 50 mM Tris HCl for pH 7.0 to 9.0 and 50 mM CAPS for pH 10.0 to 12.0. GdnHCl was dissolved in Tris 50 mM at pH 8.5 for carrying out the different experiments. The suspension of IBs was maintained at 1 bar for 16 h or placed in plastic bags that were sealed, placed inside a larger plastic bag that was vacuum-sealed and then in the pressure vessel (R4-6-40, High Pressure Equipment). The vessel was pressurized at 2.4 kbar using suitable high-pressure pump (PS-50, High Pressure Equipment) and oil as a transmission fluid, incubating then under this condition for 90 minutes. The decompression was performed slowly to 0.4 kbar, maintaining then under this condition for 14h 30min. The samples were centrifuged at 12,000 x g for 15 minutes to remove insoluble aggregates and dialyzed overnight against 50 mM TrisHCl buffer at a pH of 8.5 to lower the pH and for removal of additives. The solution was centrifuged again and stored at -20 °C for further analysis.

### Fluorescence and light scattering (LS)

Fluorescence and LS measurements were performed on a Cary Eclipse (Varian) spectrofluorimeter. Data were collected using 1 cm optical path cuvettes and measures were performed at a 90° angle relative to the incident light, using a 1 second response time and reading speed of 240 nm/minute. LS measurements were performed with excitation at 320 nm, and scattering was measured from 315 to 325 nm. The intrinsic fluorescence emission of tryptophan (Trp) was measured between 300 and 400 nm, with excitation at 290 nm.

### Polyacrylamide gel electrophoresis (SDS-PAGE)

SDS-PAGE was performed using 12% gel containing SDS, stained with Coomassie Blue G-250 [17]. For protein analysis under reducing condition, the sample buffer also contained 1 mM dithiothreitol (DTT). The intensity of the bands of NS1, used for obtaining the percentage of refolded NS1 in relation to the total protein present in the IBs suspension, was determined with basis on the “Image J” program.

### Size exclusion chromatography (SEC) analysis

Analysis of NS1 samples of DENV were performed on a Superdex 200 10/300 (GE Healthcare) column coupled to an AKTA (GE Healthcare) system. The buffer used for elution was Tris 50 mM at a pH of 8.5 or CAPS 50 mM at a pH of 11.0. Ovalbumin monomer (44.3 kDa), dimer (88.6 kDa), trimer (132.9 kDa) and bovine serum albumin monomer (66.4 kDa) were used to calibrate the Superdex 200 10/300 column. The equation: Y_VE_ = -0.027X_MW_ + 14.20 was obtained, where VE is elution volume and MW is molecular mass.

### Enzyme-Linked Immunosorbent Assay (ELISA)

Positive and negative human sera for DENV antibodies were collected under the approval of the Ethics Committee (CEPSH—Off.011616) of the Institute of Biosciences of the University of São Paulo. For 96-well plaque sensitization, 100 μL of NS1 diluted to 2 ng/ μL in a pH 7.2 phosphate saline buffer (PBS) was applied to each well and the plates were incubated at 4 °C for 16 to 18 hours. The plates were blocked with 200 μL/well of PBS containing 0.05% Tween 20, 3% milk and 0.5% BSA (blocking and dilution buffer) for 2 h at 37 °C. The blocking solution was discarded and 100 μL of serum from patients, positive or negative for DENV antibodies, diluted in dilution buffer were added to each well and incubated at room temperature for 1 hour. The wells were washed 3 times with 300 μL of wash buffer and 100 μL of peroxidase-conjugated goat anti-human IgG antibody (A0170, Sigma Aldrich), diluted 3,000-fold in dilution buffer, were added to each well. The plates were incubated again (37 °C, 1h) and washed 3 more times with wash buffer. The reaction was performed by the addition of the substrate, 0.04% of o-phenylenediamine (OPD) and 0.04% hydrogen peroxide in 100 μL of 33 mM citrate-phosphate buffer at a pH 5.5 to each well and incubated in the dark for 15 minutes at room temperature. The reaction was blocked by the addition of 50 μL of 1 M sulfuric acid. The plates were read in a spectrophotometer (Labsystems Multiscan, Thermoscientific, USA) at 492 nm.

## Results and discussion

### Solubilization of NS1-IB at HHP and high pH

**Fig 1:**
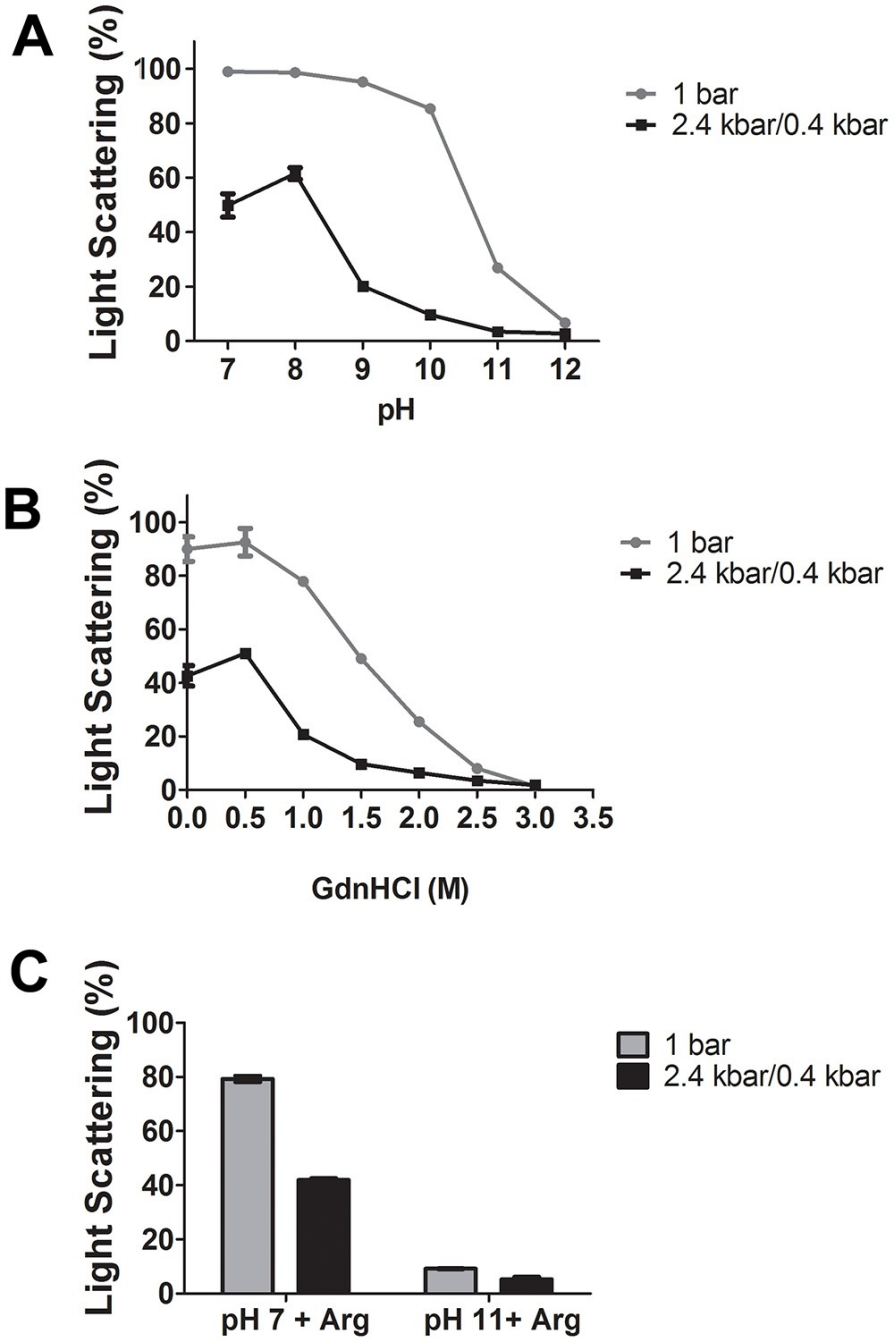
Incubation at HHP promotes solubilization of NS1-IBs. Suspensions of NS1-IBs were subjected to 2.4 kbar for 90 min and to 0.4 kbar for 14 h30 min (2.4/0.4 kbar) or to 1 bar for 16 h. **A**, LS vs pH; **B**, LS vs GdnHCl concentration at pH 8.5. The LS value, obtained for the suspension at a pH 7.0 that was not subjected to HHP and read immediately after dilution, was considered to be 100%. **C**, LS vs pH and presence of Arg. The LS value obtained for the suspension at pH 7.0, determined after 16 h incubation, was considered to be 100%. The LS measurements were carried out in a spectrofluorimeter with an excitation of 320 nm; the emission was determined between 315 and 325 nm and the areas of the peaks were used for plotting. Values are expressed as LS mean ± SD. Each condition was analysed in quadruplicate. The results shown are representative of 3 independent assays.

With the aim to solubilize IBs of a recombinant form of the dengue virus NS1 protein expressed in *E*.*coli* (NS1-IB) as mildly as possible, we used the combinations of HHP and alkaline pH as well as HHP and GdnHCl. The conditions of application of HHP were based on Malavasi and cols [18], in which we had previously shown that Green fluorescent protein (GFP) IBs were solubilized by application of 2.4 kbar, but that refolding of this protein, monitored via green fluorescence formation, occurred by incubation at lower pressure levels (0.35–0.69 kbar). Incubation at 0.4 kbar gave, moreover, the advantage of preventing reaggregation. The solubilization of NS1-IBs subjected to HHP for 16 h (2.4 kbar for 90 min and 0.4 kbar for 14h30 min) or incubated at 1 bar for the same period was monitored using visible LS. Solubilization of the IBs at HHP, as shown by the lower values of LS, is more efficient than incubation at 1 bar (Fig 1 and S1 Table). The solubilization efficiency was enhanced at alkaline pH (Fig 1A). At a pH of 8.5, incorporation of GdnHCl was required for complete solubilization of NS1-IBs, even at HHP (Fig 1B). The amino acid Arg, that generates an alkaline pH, was described to solubilize aggregated proteins and to suppress protein aggregation [19, 20]. The presence of Arg and pH 7 at 1 bar, however, only slightly solubilized NS1-IBs, while nearly complete solubilization was obtained at the Arg natural pH (11.0), for both pressure conditions (Fig 1C), which suggest that the most important factor for NS1-IBs solubilization is the alkaline pH, rather than the presence of Arg.

**Fig 2:**
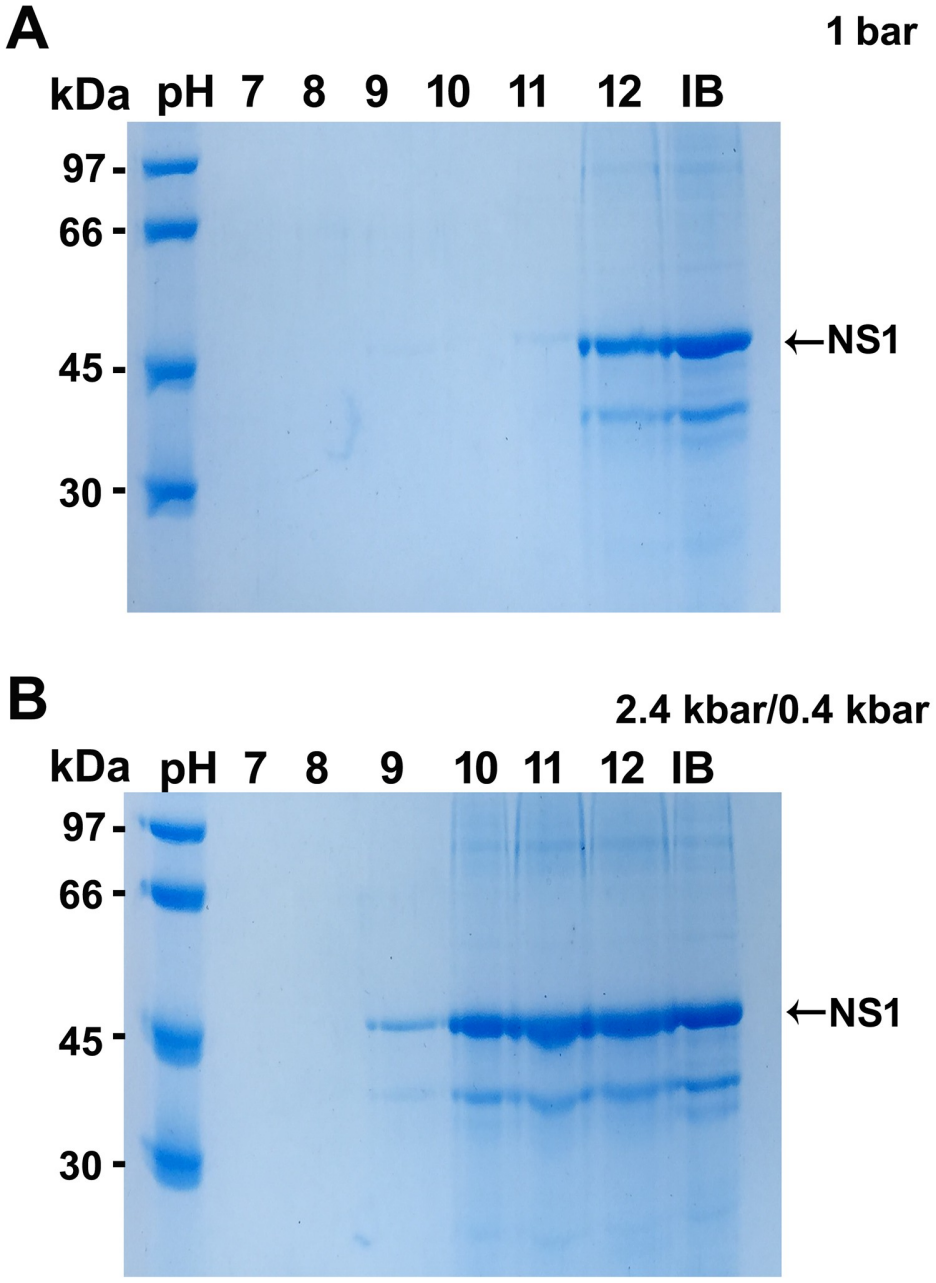
NS1 is found in the soluble fraction of the samples subjected to HHP at alkaline pH. **A**, SDS-PAGE analysis of the supernatant of the suspension incubated at 1 bar at different pH; **B**, SDS-PAGE analysis of the supernatant of the suspension incubated at HHP (2.4 kbar for 90 min and 0.4 kbar for 14h 30 min) at different pH. The results shown are representative of 3 independent assays.

NS1 monomers were present in the soluble fractions of NS1-IBs subjected to HHP and alkaline pH ranging from 10 to 12 (Fig 2), while at 1 bar NS1 solubilization only occurred at pH 12.0. Collectively, these results show that association of HHP and alkaline pH promote the efficient solubilization of dengue virus NS1. It is likely that IBs solubilization occurs by rupture of intermolecular hydrophobic interactions induced by HHP aided by the disruption of polar interactions induced by electrostatic repulsion under alkaline conditions [8, 11]. In addition to the band of 45 kDa, another band of approximately 35 kDa can also be observed in the IBs control, as well as in the solubilized samples in Fig 2. A similar band was also described by other authors for DENV NS1 that report it as a NS1 degradation product [21, 22] or to artifacts generated by an anomalous electrophoretic behavior of the NS1 protein [16].

### Analysis of NS1 solubilized by HHP and alkaline pH

**Fig 3:**
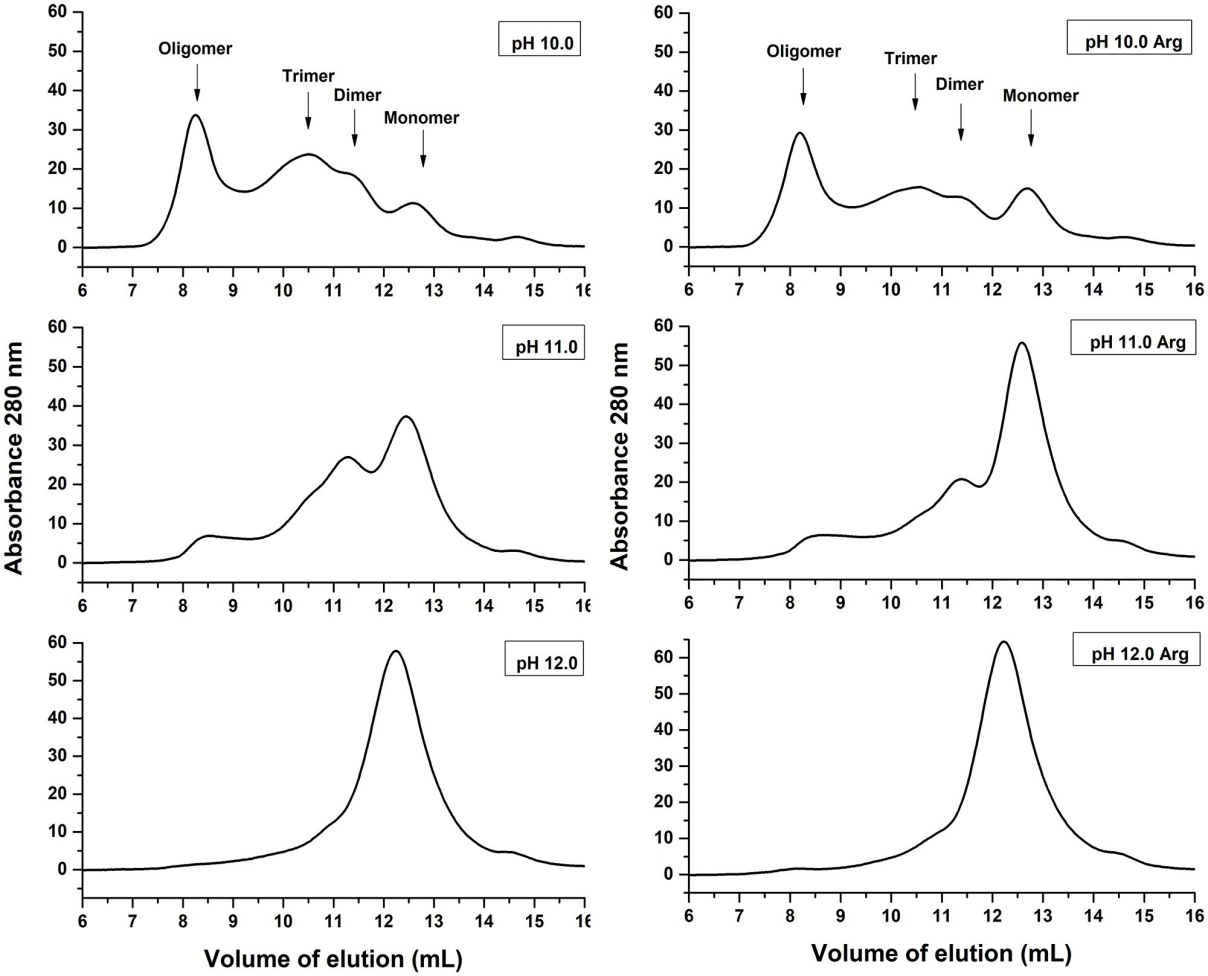
Application of HHP at pH 11.0–12.0 dissociate NS1-IBs oligomer*s*. SEC of supernatants of DENV NS1-IB suspensions subjected to 2.4 kbar/0.4 kbar. A volume of 500 μl of the supernatants of the suspensions subjected to HHP was applied to a Superdex 200 10/300 column (GE Biosciences). The elution buffer was 50 mM CAPS at a pH of 11.0. The results shown are representative of 3 independent assays.

NS1 produced by eukaryotic cells is a glycosylated dimeric protein [23]. Soluble recombinant NS1 produced in *E*. *coli* strains may be found in both monomeric and multimeric forms [21]. SEC analysis was performed in order to determine the state of NS1 dissociated by HHP and alkaline pH before dialysis (Fig 3). According to the equation obtained for SEC analysis (described in the Materials and Methods section), monomeric NS1 (43.3 kDa) should elute at a volume of 13.0 mL, while NS1 dimers and trimers at volumes of 11.7 mL and 10.5 mL, respectively. We considered, therefore, the peaks at elution volumes of approximately 12.8 mL, 11.3 mL and 10.5 mL as NS1 monomer, dimer and trimer respectively. The peak with higher intensity observed in the supernatant of NS1-IBs that was subjected to HHP at a pH of 10.0 is the one that eluted at a volume 8.3 mL, which is in a position of oligomers of NS1 presenting more than 500 kDa. The presence of Arg and pH increase promoted a shift of the peak to higher elution volumes, indicating dissociation of NS1-IBs into forms presenting lower molecular weights. The presence of NS1 oligomers has been reported [23]. Application of HHP at a pH of 11.0 induced the dissociation of aggregates mostly into dimer and monomer, while monomer was found almost exclusively after solubilization at pH 12.0. Summarizing, higher pH and the presence of Arg induces a more effective dissociation of NS1 into monomer and dimer.

### Determination of NS1 unfolding by HHP treatment

**Fig 4:**
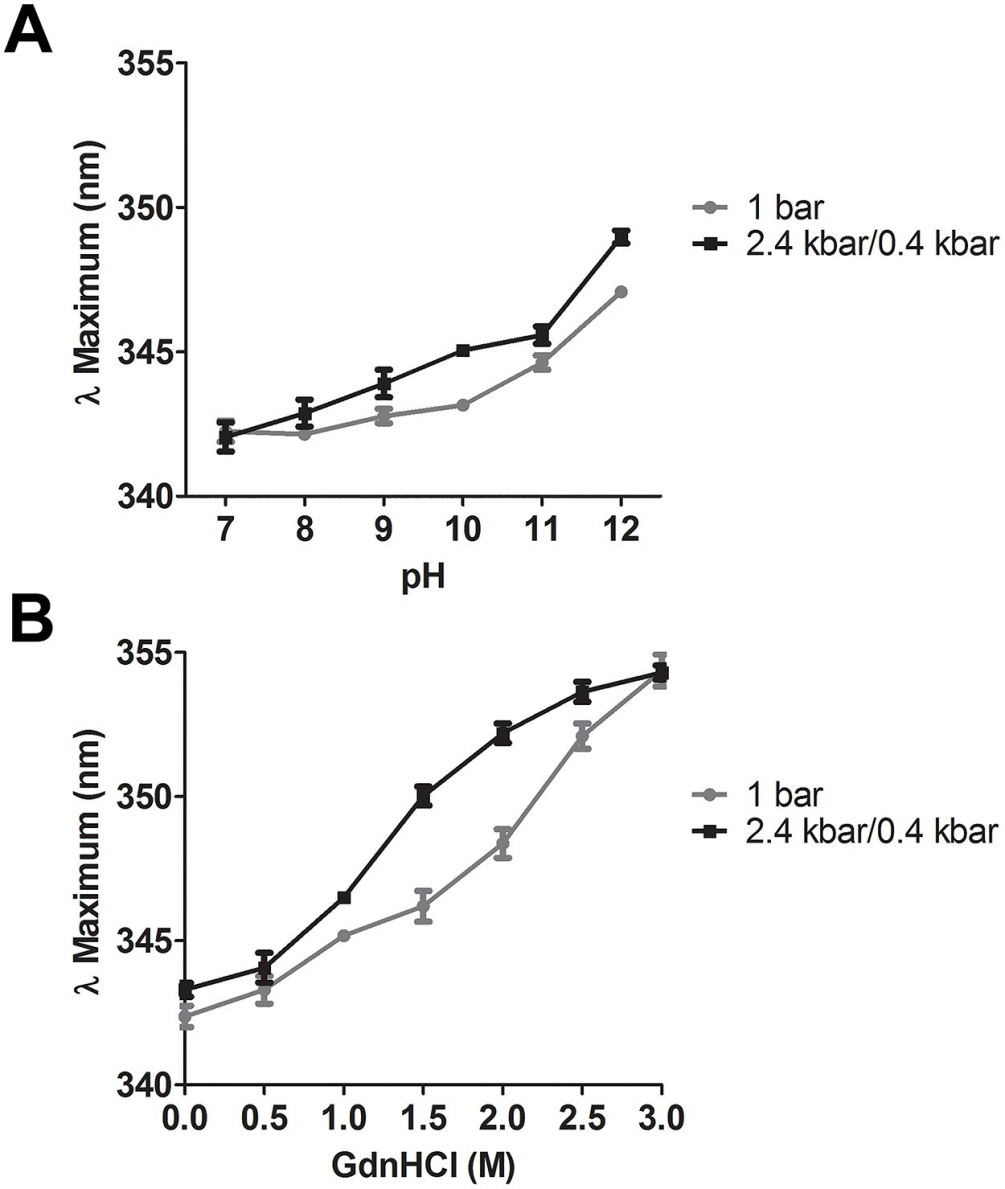
HHP in association with alkaline pH and the presence of Arg induce only partial NS1 unfolding. Suspensions of NS1-IB were subjected to 2.4 kbar/0.4 kbar or to 1 bar treatment. **A**, **λ** maximum vs pH; **B**, **λ** maximum vs GdnHCl concentration. An excitation at 290 nm was used for intrinsic fluorescence determination and the emission was measured between 300 and 400 nm. Each condition was analysed at least in triplicate. Values are expressed as mean ± SD. The results shown are representative of 3 independent assays.

In order to evaluate the maintenance of NS1 secondary and tertiary structures that are frequently found in IBs, we monitored the effect of NS1-IBs treatments on protein unfolding. A peak of Trp fluorescence with maximal intensity (**λ** maximal) at 344 nm was described for DENV NS1 while a shift of 11 nm towards higher wavelength (355 nm), which is indicative of exposure of the Trp to a more hydrophilic environment and protein unfolding, was observed for the denatured protein [21] and also in the present study. At a pH of 7.0, we obtained a **λ** maximal of 342.2 nm for NS1-IBs. An increment of pH induced small shifts towards higher wavelengths indicating increased unfolding of NS1. Nonetheless, NS1 subjected to HHP showed a slightly higher degree of unfolding than samples prepared at 1 bar, with short shifts to higher wavelength of approximately 1.0–2.0 nm (Fig 4A).

We had shown in Fig 1 that NS1-IBs subjected to HHP at pH of 10 showed a similar degree of solubilization than the protein submitted to HHP at a pH of 8.5 in the presence of 1.5 M GdnHCl. Nonetheless, as indicated in Fig 4A and 4B and S2 Table, NS1 solubilized at pH 10 and 11 showed **λ** maximum of 345.1 nm and 345.6 nm respectively, with lower degree of unfolding (**λ** maximal shifts of 2.9 nm and 3.4 nm in relation to non-treated NS-IBs) than the protein solubilized at pH 8.5 in the presence of 1.5 M GdnHCl, with a maximal **λ** at 350.0 nm (shift of 7.8 nm). These results indicate that association of HHP with pH 10–11 represents a milder alternative for solubilization of IBs in comparison with the association of HHP in the presence of the chaotropic reagent; at pH 12, however, the level of unfolding was higher (**λ** maximal shift of 6.9 nm).

### Refolded NS1 analyzed by SEC

**Fig 5:**
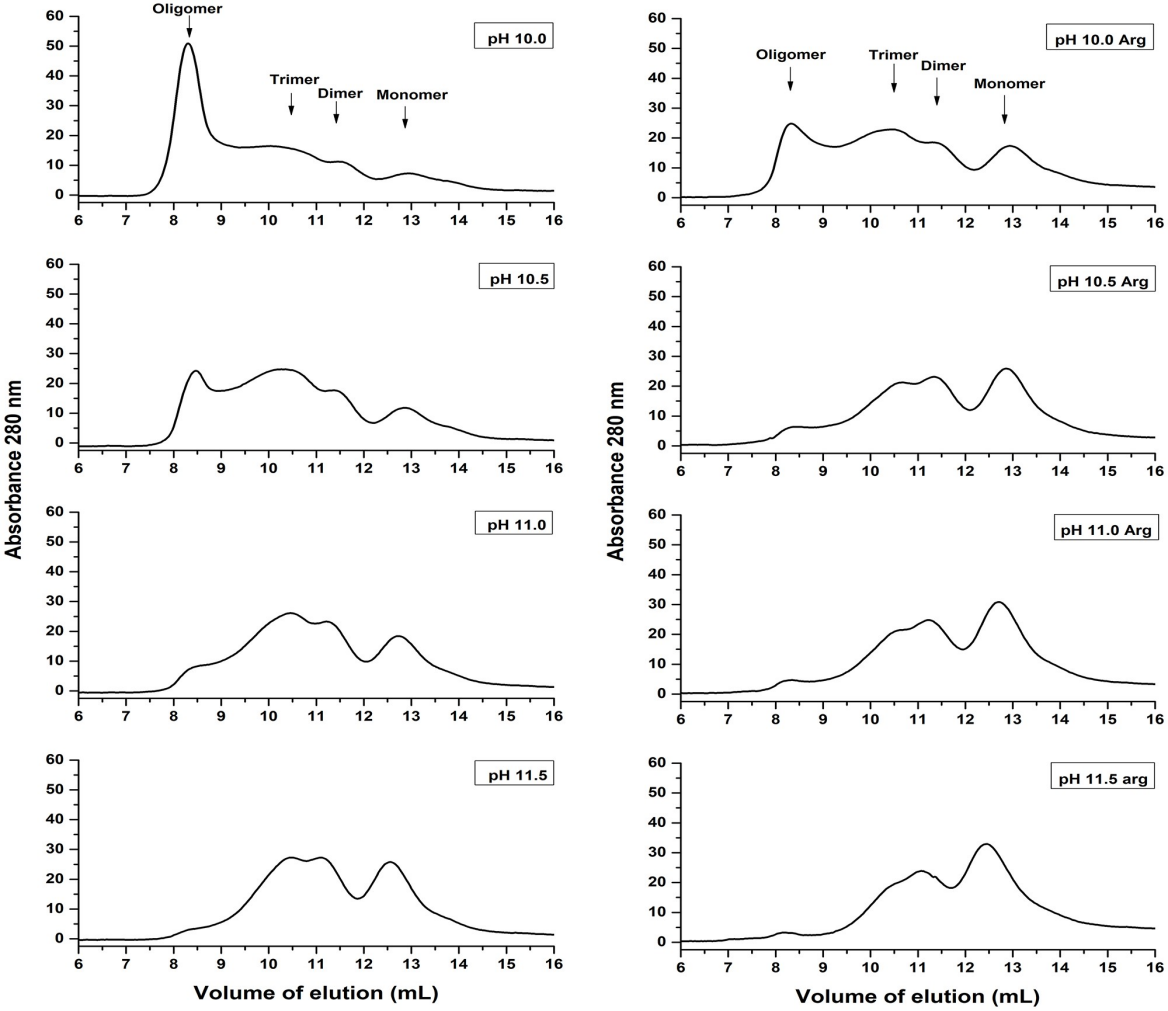
NS1 solubilized by application of HHP at pH of 10.0–11.5 and dialyzed at a pH of 8.5 forms mostly trimers, dimers and monomers. DENV NS1-IB suspensions were subjected to 2.4 kbar/0.4 kbar and dialyzed against 50 mM Tris HCl at a pH of 8.5. A volume of 500 μl of the supernatant of the suspensions was applied to a Superdex 200 10/300 column (GE Biosciences). The elution buffer was TrisHCl 50 mM at a pH of 8.5. The results shown are the representative of 2 independent assays.

The refolding of NS1 solubilized at HHP and alkaline pH was obtained by dialysis to a lower pH (8.5). SEC analysis of the refolded NS1 was performed with the objective to determine if NS1 re-associates to higher molecular weight forms by dialysis (Fig 5). The presence of oligomers was observed in samples subjected to HHP at pH 10.0, as expected, based on the fact that the application of HHP at this pH was not effective for NS1 oligomers dissociation. However, a reduction in the oligomers was found for the samples subjected to HHP at pH 10.5 and in the presence of Arg. As indicated in the chromatograms shown in Fig 5 for the sample subjected to HHP at pH 10.5 in the presence of Arg, we observed that 5.8% of NS1 was present in oligomeric form (6.36 mg/liter), 29.8% as trimer (32.7 mg/liter), 22.9% (25.1 mg/liter) as dimer and 41.5% (45.5 mg/liter of bacterial culture) as monomer. Based on these results, the condition chosen to solubilize NS1 for subsequent refolding was pH 10.5, in the absence or in the presence of Arg.

### Yield of NS1 refolding

**Fig 6:**
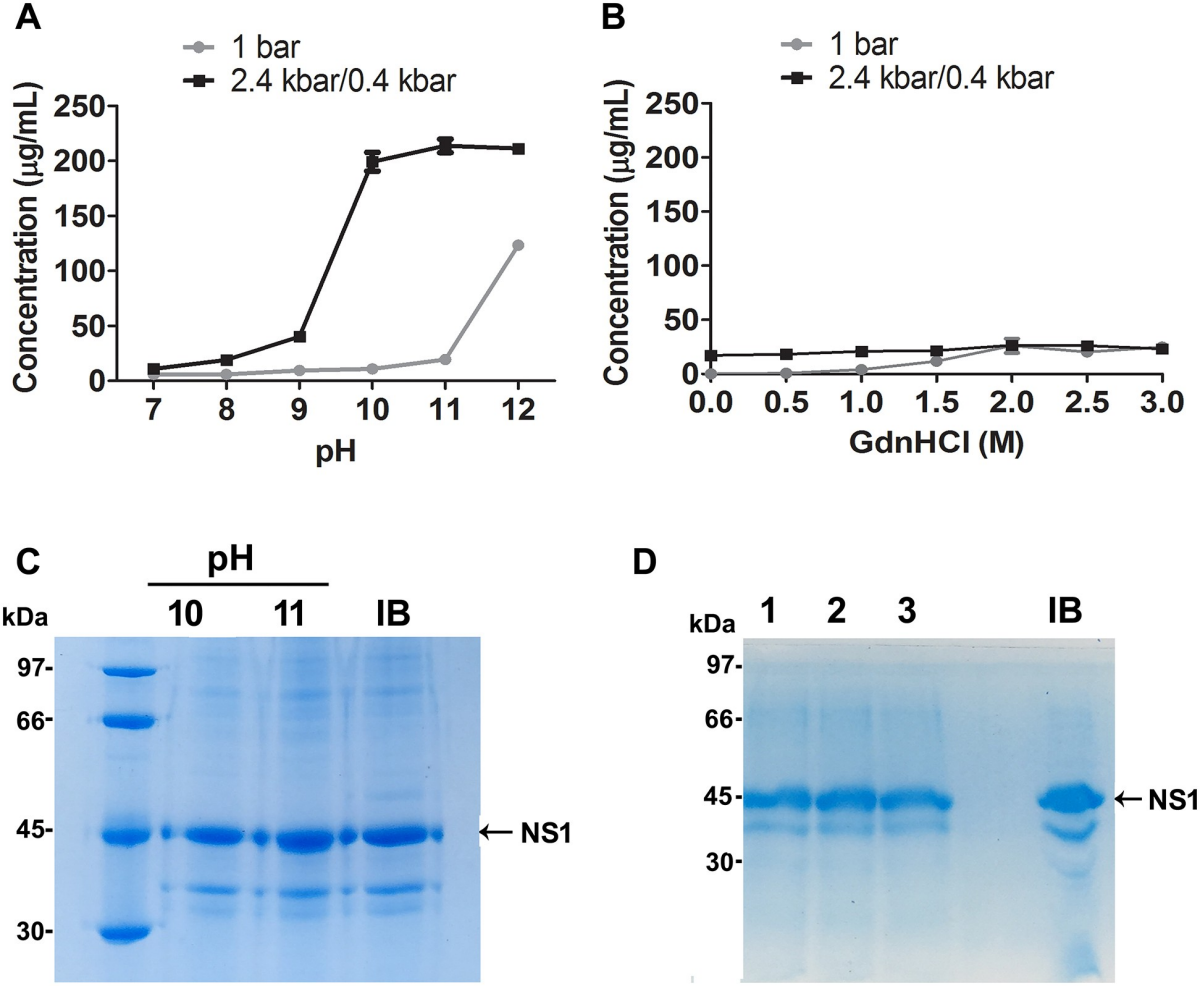
Concentration of NS1 in the supernatants of NS1-IB subjected to HHP. Suspensions of NS1-IB were subjected to 2.4 kbar/0.4 kbar or to 1 bar. **A)** NS1 concentration vs pH; **B**) NS1 concentration vs GdnHCl concentration. Values are expressed as LS mean ± SD. Each condition was analysed at least in triplicate and the assay was performed 4 times. **C)** NS1 refolded at HHP and the pH indicated; D, NS1 refolded at HHP. Column 1, pH 10.5 + 0.4 M arg, column 2, pH 11.0 + 0.4 M Arg, column 3, pH 11.0 + 0.4 M Arg + 1 mM GSH + 0.1 mM GSSG. IB, NS1-IB suspension. The results shown are representative of 6 independent assays.

The amount of NS1 in the supernatant of the samples subjected to HHP at pH 10–12 was 8-fold higher than the amount found in the samples that were subjected to HHP in the presence of GdnHCl at a pH of 8.5 (Fig 6A and 6B and S3 Table), a strategy similar to that already described for the refolding of other proteins using HHP [9, 24–27]. The low concentration of NS1 in the samples solubilized in the presence of GdnHCl is probably associated with protein aggregation after removal of the chaotropic reagent during dialysis. This result confirms that HHP in association with alkaline pH is the most appropriate condition for NSI-IB solubilization. NS1 compressed at pH 10 and 11 is almost pure with the exception of a small amount of the 35 kDa band already shown in Fig 2 (Fig 6C). The refolding experiments were performed 3 times and the yield of refolded NS1 for the samples subjected to HHP at pH 10.5 in the presence of Arg were similar to the samples compressed at pH 11 in the presence or in the absence of Arg: 93.2 ± 14.1 mg/L bacterial culture at a relatively high concentration (up to 0.4 mg NS1/mL). A mixture of low molecular weight thiol and disulfide-containing compounds, known as disulfide shuffling agents, such as reduced and oxidized glutathione (GSH/GSSG) are commonly added to refolding buffers to allow disulfide bond formation and shuffling [28]. The presence of Arg in association with the disulfide shuffling agents GSH (1 mm) and GSSG (0.1 mM) slightly improved the volumetric yield of NS1 refolding to 109 mg/L ± 9.5 mg/L bacterial culture (Fig 6D), which is possibly related to an improvement in folding due to correct disulfide bond formation. This yield is more than 30-fold higher than the one obtained by Amorim and cols for the refolding of NS1-IBs generated by the same bacterial clone, using an established protocol at atmospheric pressure: 3.5 mg/L [16]. The refolding yield of NS1, obtained by Image J program analysis of the SDS-PAGE bands, as described in Materials and Methods, is very high: 90–94% of the total protein present in IBs (Fig 6D).

### Reactivity of DENV-positive human serum with refolded NS1

**Fig 7:**
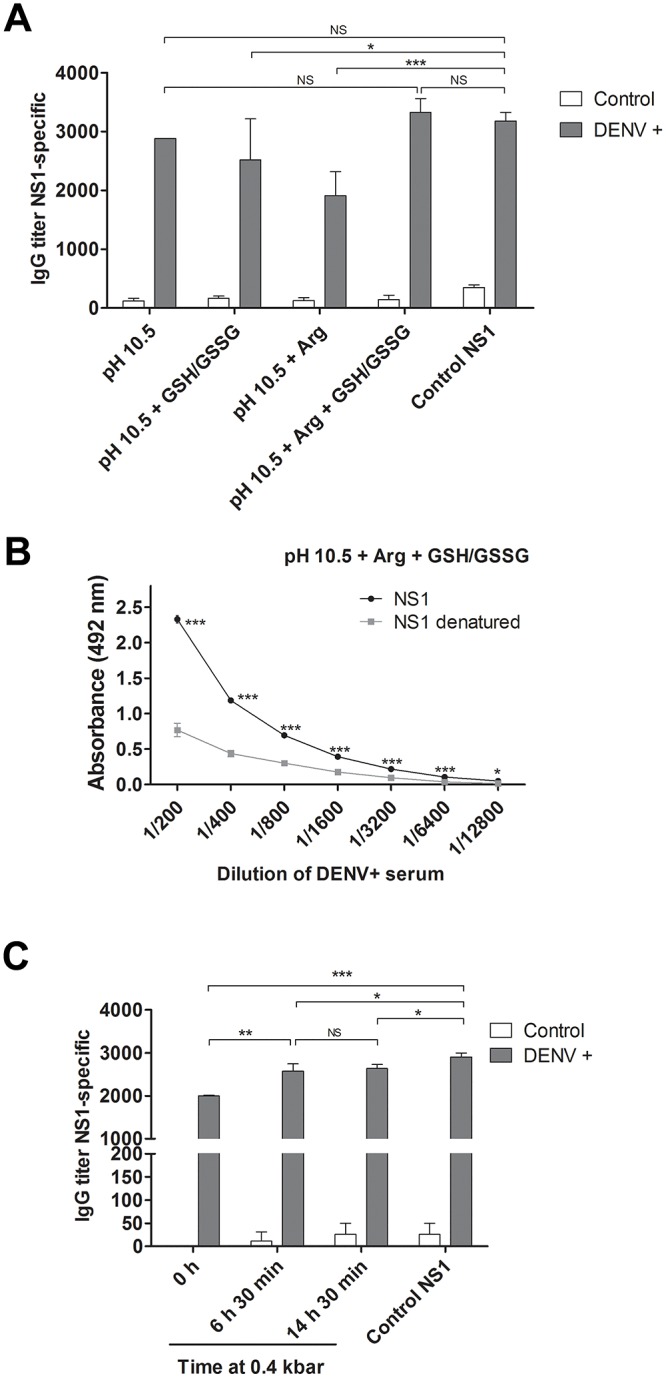
DENV NS1 refolded at HHP is antigenic. **A)** Titers of NS1-specific IgG measured by ELISA using NS1 refolded with HHP at pH 10.5 in different conditions. The ELISA was employing with a control sera obtained from patients that had been previously infected with DENV (gray bars) or not (white bars). **B)** Evaluation of the preservation of conformational epitopes in NS1. NS1 obtained at HHP, pH 10.5 + Arg + GSH/GSSG was previously denatured (100 °C, 10 min) or not and analyzed by ELISA for reactivity with a serum from a patient previously infected with DENV. For A and B, the IB compression was performed in 2.4 kbar / 0.4 kbar. **C)** Titers of NS1 refolded at HHP at pH 10.5 + Arg + GSH/GSSG by incubation at 2.4 kbar (90 min.) and 0.4 kbar for the time indicated in the figure. Values are expressed as mean ± SD of the data. All NS1 samples had low reactivity with control serum in ELISA. Control NS1, obtained from the same clone used in this study and refolded using traditional protocols performed at atmospheric pressure. * p < 0.05, ** p <0.01, *** p<0.001 (Two way ANOVA with Bonferroni post-test). The results shown are the representative of 3 independent assays.

The NS1 protein obtained with HHP at pH 10.5, in the presence or absence of the Arg/GSH/GSSH combination, preserved its antigenicity, as measured by ELISA using a serum sample from patient previously infected by DENV. Reduced NS1-specific IgG titers, however, were obtained when the refolding procedure was performed in presence of the Arg or GSH/GSSH additives separately (Fig 7A and S4 Table). As expected, all IgG titers were significantly reduced when the NS1 protein was reacted with a control sera obtained from a non-infected individual.

To assess whether the NS1 protein, obtained in the presence of Arg/GSH/GSSH combination, preserved conformational epitopes in its structure, this protein was reacted in the native or denatured forms with a DENV+ sera by ELISA. Lower absorbance values were moreover obtained after heat denaturing of NS1 (Fig 7B and S4 Table), suggesting that the refolded protein preserves conformational epitopes of the native viral protein. Moreover, higher titers of NS1-specific IgG were obtained for NS1 refolded by incubation at 2.4 kbar for 90 min followed by incubation at 0.4 kbar (6 h 30 min or for 14 h 30 min) than for the protein incubated only at 2.4 kbar. This suggests that NS1 folding is favored during incubation at an intermediary pressure (Fig 7C and S4 Table), indicating that the process can be completed in less than 16 h. We cannot forget, however, that immunoreactivity of a protein is not strictly synonymous of properly refolded 3D structure, since antibodies may react to a small poly-peptide fraction which is not necessarily in the native folded state.

## Conclusions

The utilization of HHP concomitantly with alkaline pH allowed the use of a pH lower that it would be necessary to efficiently solubilize the aggregates at atmospheric pressure, with the benefit of avoiding the utilization of chaotrope reagents. It does not induce important loss of NS1 tertiary structure and was highly efficient to promote the dissociation of NS1-IBs to dimers and monomers.

The refolding process using the association of HHP and alkaline pH is based on only two steps: solubilization of IBs via HHP and dialysis. The whole process is very quick and easy, being completed in less than 24 hours. In addition, the protein concentration during compression and dialysis can be relatively high (up to 0.4 mg NS1 / mL) and the refolded protein present high purity. This innovative process can be a very interesting alternative also for the refolding of other proteins of interest.

## Supporting information

S1 Table Fig 1 Dataset Table (LS). A, LS vs pH; B, LS vs GdnHCl concentration and C, LS vs pH in the absence or in the presence of Arg.
(DOCX)

S2 Table Fig 4 Dataset Table (λ maximum). A, λ vs pH and B, λ vs GdnHCl concentration.
(DOCX)

S3 Table Fig 6 Dataset Table (NS1 concentration). A, NS1 concentration vs pH and B, NS1 concentration vs GdnHCl concentration.
(DOCX)

S4 Table Fig 7 Dataset Table (ELISA). A, Titres of NS1-specific IgG. NS1 refolded at HHP and pH 10.5 and different conditions; B, Evaluation of preservation of NS1 conformational epitopes; C, NS1 refolded by incubation at 0.4 kbar for different times.
(DOCX)

## Abbreviations

CAPS: 3-(Cyclohexyl)-1-propanesulfonic acid
DENV: dengue virus
DTT: dithiothreitol
EDTA: ethylenediaminetetraacetic acid
ELISA: enzyme-linked immunosorbent assay
GdnHCl: guanidine hydrochloride
GSH: reduced glutathione
GSSG: oxidized glutathione
HHP: high hydrostatic pressure
IPTG: isopropyl β-D-thiogalactopyranoside
LB: Luria-Bertani broth
LS: light scattering
NS1: Non-structural protein 1
NS1-IBs: Inclusion bodies of NS1
SDS-PAGE: sodium dodecyl sulphate polyacrylamide gel electrophoresis
