# Supplementary material for: Protein refolding based on high hydrostatic pressure and alkaline pH: Application on a recombinant dengue virus NS1 protein
Source: PLoS One. 2019 Jan 25;14(1):e0211162. doi: 10.1371/journal.pone.0211162 (PMC6347194; doi:10.1371/journal.pone.0211162)
Supplement: S1 Table — A, LS vs pH; B, LS vs GdnHCl concentration and C, LS vs pH in the absence or in the presence of Arg. (DOCX) [file pone.0211162.s001.docx]

**S1 Table**

**Dataset Figure 1. Light scattering**

1. **LS vs pH**

| **pH** | **1 bar** | | | | **Mean** | **SD** | **2.4 kbar/0.4 kbar** | | | | **Mean** | **SD** |
| --- | --- | --- | --- | --- | --- | --- | --- | --- | --- | --- | --- | --- |
| **7** | 98.0 | 98.3 | 99.2 | 100.3 | 98.95 | 1.034 | 46.2 | 48.7 | 56.0 | 48.3 | 49.8 | 4.276 |
| **8** | 100.5 | 98.6 | 97.0 | 98.3 | 98.60 | 1.444 | 60.6 | 61.9 | 64.3 | 59.2 | 61.5 | 2.168 |
| **9** | 94.1 | 95.5 | 94.0 | 96.9 | 95.12 | 1.367 | 18.8 | 21.7 | 20.4 | 20.0 | 20.2 | 1.195 |
| **10** | 82.7 | 87.1 | 85.5 | 86.0 | 85.32 | 1.873 | 11.0 | 8.9 | 9.3 | 9.6 | 9.7 | 0.912 |
| **11** | 26.4 | 25.8 | 26.5 | 28.8 | 28.87 | 1.320 | 3.2 | 3.3 | 3.6 | 4.0 | 3.5 | 0.359 |
| **12** | 6.8 | 6.7 | 7.1 | 6.4 | 6.750 | 0.288 | 2.4 | 2.8 | 2.9 | 3.0 | 2.7 | 0.263 |

1. **LS v GdnHCl concentration**

| **GdnHCl (M)** | **1 bar** | | | | **Mean** | **SD** | **2.4 kbar/0.4 kbar** | | | | **Mean** | **SD** |
| --- | --- | --- | --- | --- | --- | --- | --- | --- | --- | --- | --- | --- |
| **0** | 83.2 | 93.0 | 92.1 | 91.5 | 89.95 | 4.542 | 39.0 | 46.2 | 39.6 | 45.6 | 42.60 | 3.826 |
| **0.5** | 85.4 | 95.6 | 96.9 | 92.2 | 92.52 | 5.146 | 50.6 | 50.0 | 52.5 |  | 51.03 | 1.305 |
| **1.0** | 77.0 | 78.7 | 77.6 | 78.3 | 77.90 | 0.752 | 21.8 | 21.7 | 18.7 |  | 20.73 | 1.761 |
| **1.5** | 47.9 | 48.4 | 51.2 | 48.7 | 49.05 | 1.470 | 10.4 | 10.0 | 8.0 | 10.4 | 9.70 | 1.148 |
| **2.0** | 23.4 | 26.2 | 25.3 | 26.8 | 25.42 | 1.484 | 8.4 | 5.3 | 5.9 | 6.1 | 6.42 | 1.359 |
| **2.5** | 8.1 | 8.3 | 7.3 | 8.2 | 7.97 | 0.457 | 3.4 | 4.5 | 3.3 | 2.8 | 3.50 | 0.716 |
| **3.0** | 1.7 | 1.3 | 1.4 | 1.0 | 1.35 | 0.288 | 2.0 | 1.7 | 1.7 | 1.7 | 1.77 | 0.150 |

1. **LS vs pH in the absence or in the presence of Arg**

| **LS (%)** | **1 bar** | | | **Mean** | **SD** | **2.4 kbar/0.4 kbar** | | | **Mean** | **SD** |
| --- | --- | --- | --- | --- | --- | --- | --- | --- | --- | --- |
| **LS% pH 7 + Arg** | 79.2 | 78.1 | 80.5 | 79.26 | 1.201 | 41.6 | 42.7 | 41.6 | 41.96 | 0.635 |
| **LS% pH 11 + Arg** | 9.0 | 9.1 | 9.4 | 9.16 | 0.208 | 5.9 | 5.7 | 4.2 | 5.26 | 0.929 |
