# Supplementary material for: Protein refolding based on high hydrostatic pressure and alkaline pH: Application on a recombinant dengue virus NS1 protein
Source: PLoS One. 2019 Jan 25;14(1):e0211162. doi: 10.1371/journal.pone.0211162 (PMC6347194; doi:10.1371/journal.pone.0211162)
Supplement: S2 Table — A, λ vs pH and B, λ vs GdnHCl concentration. (DOCX) [file pone.0211162.s002.docx]

**S2 Table**

**Dataset Figure 4. λ maximal (nm)**

1. **λ maximum vs pH**

| **pH** | **1 bar** | | | | **Mean** | **SD** | **2.4 kbar/0.4 kbar** | | | | **Mean** | **SD** |
| --- | --- | --- | --- | --- | --- | --- | --- | --- | --- | --- | --- | --- |
| **7** | 341.8 | 342.7 | 342.2 | 342.3 | 342.25 | 0.37 | 341.7 | 342.7 | 341.6 | 342.2 | 342.05 | 0.506 |
| **8** | 342.2 | 342.2 | 342.1 | 342.1 | 342.15 | 0.06 | 343.5 | 342.4 | 342.7 | 342.9 | 342.88 | 0.464 |
| **9** | 342.9 | 342.4 | 342.9 | 342.9 | 342.77 | 0.25 | 343.6 | 343.6 | 344.6 | 343.8 | 343.90 | 0.476 |
| **10** | 343.0 | 343.0 | 343.3 | 343.3 | 343.15 | 0.17 | 345.2 | 344.8 | 345.2 | 345.0 | 345.05 | 0.191 |
| **11** | 344.6 | 344.9 | 344.3 | 344.7 | 344.62 | 0.25 | 345.5 | 345.5 | 345.3 | 346.0 | 345.58 | 0.298 |
| **12** | 347.0 | 347.1 | 347.0 | 347.2 | 347.07 | 0.09 | 349.2 | 349.1 | 348.9 | 348.7 | 348.98 | 0.221 |

1. **λ maximum vs GdnHCl concentration**

| **GdnHCl (M)** | **1 bar** | | | | **Mean** | **SD** | **2.4 kbar/0.4 kbar** | | | | **Mean** | **SD** |
| --- | --- | --- | --- | --- | --- | --- | --- | --- | --- | --- | --- | --- |
| **0** | 342.4 | 341.9 | 342.4 | 342.8 | 342.38 | 0.368 | 343.4 | 343.1 | 343.6 | 343.1 | 343.3 | 0.244 |
| **0.5** | 343.4 | 342.6 | 343.6 | 343.6 | 343.30 | 0.476 | 344.2 | 344.5 | 343.5 |  | 344.0 | 0.513 |
| **1.0** | 345.2 | 345.2 | 345.3 | 345.0 | 345.17 | 0.125 | 346.7 | 346.3 | 346.5 |  | 346.5 | 0.200 |
| **1.5** | 346.5 | 345.8 | 345.7 | 346.8 | 346.20 | 0.535 | 350.4 | 350.1 | 350.0 | 349.6 | 350.0 | 0.330 |
| **2.0** | 347.7 | 348.9 | 348.4 | 348.5 | 348.37 | 0.499 | 352.1 | 352.3 | 351.8 | 352.6 | 352.2 | 0.336 |
| **2.5** | 351.9 | 352.3 | 351.6 | 352.6 | 352.10 | 0.439 | 353.3 | 353.6 | 354.0 |  | 353.6 | 0.351 |
| **3.0** | 354.8 | 354.9 | 354.0 | 353.8 | 354.37 | 0.556 | 354.6 | 354.3 | 354.3 | 354.0 | 354.3 | 0.244 |
