# Supplementary material for: Protein refolding based on high hydrostatic pressure and alkaline pH: Application on a recombinant dengue virus NS1 protein
Source: PLoS One. 2019 Jan 25;14(1):e0211162. doi: 10.1371/journal.pone.0211162 (PMC6347194; doi:10.1371/journal.pone.0211162)
Supplement: S3 Table — A, NS1 concentration vs pH and B, NS1 concentration vs GdnHCl concentration. (DOCX) [file pone.0211162.s003.docx]

**S3 Table**

**Dataset Figure 6. NS1 concentration (μg/ml)**

1. **NS1 concentration vs pH**

| **pH** | **1 bar** | | | | **Mean** | **SD** | **2.4 kbar/0.4 kbar** | | | | **Mean** | **SD** |
| --- | --- | --- | --- | --- | --- | --- | --- | --- | --- | --- | --- | --- |
| **7** | 3.4 | 7.2 | 4.8 | 8.2 | 5.9 | 2.19 | 12.5 | 9.2 | 11.1 |  | 10.93 | 1.65 |
| **8** | 3.4 | 7.0 | 6.3 | 7.2 | 5.9 | 1.75 | 17.4 | 22.2 | 19.8 | 16.9 | 19.07 | 2.43 |
| **9** | 11.0 | 10.0 | 9.0 | 8.2 | 9.5 | 1.21 | 38.6 | 40.1 | 39.6 | 42.9 | 40.30 | 1.84 |
| **10** | 9.7 | 11.0 | 13.0 | 10.0 | 10.9 | 1.49 | 209.2 | 201.0 | 198.5 | 188.4 | 199.27 | 8.57 |
| **11** | 17.0 | 23.0 | 17.0 | 21.0 | 19.5 | 3.00 | 220.3 | 205.7 | 215.5 | 213.5 | 213.75 | 6.07 |
| **12** | 117.8 | 124.2 | 122.7 | 129.0 | 123.4 | 4.61 | 209.2 | 215.9 | 213.5 | 207.2 | 211.45 | 3.96 |

1. **NS1 concentration vs GdnHCl concentration**

| **GdnHCl (M)** | **1 bar** | | | **Mean** | **SD** | **2.4 kbar/0.4 kbar** | | | **Mean** | **SD** |
| --- | --- | --- | --- | --- | --- | --- | --- | --- | --- | --- |
| **0** | 0 | 0 | 0 | 0 | 0 | 15.9 | 15.0 | 20.8 | 17.23 | 3.12 |
| **0.5** | 0 | 2.02 | 0 | 0.67 | 1.166 | 16.4 | 20.3 | 17.9 | 18.20 | 1.96 |
| **1.0** | 1.9 | 2.41 | 7.7 | 4.01 | 3.201 | 19.3 | 20.8 | 22.7 | 20.93 | 1.70 |
| **1.5** | 11.1 | 10.6 | 13.5 | 11.73 | 1.550 | 21.7 | 22.2 | 20.8 | 21.56 | 0.70 |
| **2.0** | 25.6 | 19.80 | 32.8 | 26.06 | 6.512 | 28.5 | 25.1 | 26.1 | 26.56 | 1.74 |
| **2.5** | 18.4 | 23.70 | 18.8 | 20.30 | 2.951 | 23.2 | 28.0 | 27.1 | 26.10 | 2.55 |
| **3.0** | 23.2 | 26.10 | 25.6 | 24.96 | 1.550 | 23.1 | 21.7 | 24.6 | 23.13 | 1.45 |
