# Supplementary material for: Protein refolding based on high hydrostatic pressure and alkaline pH: Application on a recombinant dengue virus NS1 protein
Source: PLoS One. 2019 Jan 25;14(1):e0211162. doi: 10.1371/journal.pone.0211162 (PMC6347194; doi:10.1371/journal.pone.0211162)
Supplement: S4 Table — A, Titres of NS1-specific IgG. NS1 refolded at HHP and pH 10.5 and different conditions; B, Evaluation of preservation of NS1 conformational epitopes; C, NS1 refolded by incubation at 0.4 kbar for different times. (DOCX) [file pone.0211162.s004.docx]

**S4 Table**

**Dataset Figure 7. ELISA**

1. **Titres of NS1-specific IgG. NS1 refolded at HHP a pH 10.5 and different conditions.**

| **Refolding condition** | **DENV- serum** | | | **Mean** | **SD** | **DENV + serum** | | | **Mean** | **SD** |
| --- | --- | --- | --- | --- | --- | --- | --- | --- | --- | --- |
| **pH 10.5** | 73.79 | 153.29 | 136.11 | 121.06 | 41.83 | 2884.46 | 2878.55 | 2878.96 | 2880.65 | 3.3 |
| **pH 10.5 + GSH/GSSG** | 210.36 | 143.58 | 139.66 | 164.53 | 39.73 | 3321.24 | 2181.62 | 2053.44 | 2518.76 | 697.9 |
| **pH 10.5 + Arg** | 66.46 | 152.57 | 160.98 | 126.67 | 52.31 | 2124.44 | 1434.57 | 2170.68 | 1909.89 | 412.3 |
| **pH 10.5 + Arg + GSH/GSSG** | 62.34 | 155.90 | 211.17 | 143.13 | 75.23 | 3593.21 | 3144.58 | 3233.37 | 3323.72 | 237.6 |
| **Control NS1** | 391.99 | 345.16 | 305.09 | 347.41 | 43.49 | 3348.44 | 3073.68 | 3113.52 | 3178.54 | 148.5 |

1. **Evaluation of preservation of conformational epitopes in NS1.**

| **Serial dilution** | **NS1** | | | **Mean** | **SD** | **NS1 denatured by heat** | | | **Mean** | **SD** |
| --- | --- | --- | --- | --- | --- | --- | --- | --- | --- | --- |
| **1/200** | 2.257 | 2.425 | 2.305 | 2.329 | 0.086 | 0.783 | 0.669 | 0.854 | 0.769 | 0.093 |
| **1/400** | 1.178 | 1.149 | 1.234 | 1.187 | 0.043 | 0.408 | 0.414 | 0.489 | 0.437 | 0.045 |
| **1/800** | 0.667 | 0.671 | 0.738 | 0.693 | 0.039 | 0.286 | 0.284 | 0.334 | 0.302 | 0.028 |
| **1/1600** | 0.373 | 0.388 | 0.418 | 0.393 | 0.023 | 0.162 | 0.161 | 0.203 | 0.176 | 0.024 |
| **1/3200** | 0.211 | 0.214 | 0.230 | 0.218 | 0.010 | 0.081 | 0.090 | 0.115 | 0.096 | 0.018 |
| **1/6400** | 0.101 | 0.101 | 0.118 | 0.107 | 0.010 | 0.028 | 0.032 | 0.052 | 0.038 | 0.013 |
| **1/12800** | 0.050 | 0.047 | 0.056 | 0.051 | 0.005 | 0.008 | 0.011 | 0.024 | 0.015 | 0.009 |

1. **NS1 refolded by incubation at 0.4 kbar for different times.**

**DENV(+) serum**

| **Time** | **NS1** | | | **Mean** | **SD** |
| --- | --- | --- | --- | --- | --- |
| **0 h** | 2007.34 | 1959.72 | 2026.57 | 1997.8 | 34.41 |
| **8 h** | 2239.98 | 2765.84 | 2721.48 | 2575.7 | 291.64 |
| **16 h**  **Control** | 2528.42  2781.95 | 2818.13  2853.92 | 2550.09  3080.28 | 2632.2  2905.4 | 161.37  155.68 |

**DENV(-) serum**

| **Time** | **NS1** | | | **Mean** | **SD** |
| --- | --- | --- | --- | --- | --- |
| **0 h** | 0.06 | 0.04 | 1.59 | 0.563 | 0.89 |
| **8 h** | 0.02 | 0.00 | 34.23 | 11.41 | 19.75 |
| **16 h**  **Control** | 48.34  35.69 | 0.22  14.11 | 29.04  1.05 | 25.86  16.95 | 24.21  17.49 |
